# Gastric adenocarcinoma of the fundic gland type: A case report

**DOI:** 10.1097/MD.0000000000020361

**Published:** 2020-05-22

**Authors:** Xue Fan, Xue-Song Yang, Peng Bai, Yu-Bo Ren, Lei Zhang, Xin Li, Li Wang, Yan Wang, Yi-Ming Ding, Ran-Ran Zeng, Xiang-Chun Lin

**Affiliations:** a Department of Gastroenterology; b Department of Pathology, Peking University International Hospital, Beijing 102206, China.

**Keywords:** depressed lesion, endoscopic submucosal dissection, gastric cancer, hyperplasia

## Abstract

**Introduction:**

Gastric adenocarcinoma of the fundic gland type (GA-FG) is a newly described entity that is characterized by well-differentiated neoplasm with unclear etiopathogenesis.

**Patient concerns:**

A 60-year-old Chinese man was referred to our hospital for abdominal distension.

**Diagnosis:**

Esophagogastroduodenoscopy (EGD) showed a depressed lesion found using in the greater curvature of the stomach. The pathological diagnosis of the biopsy specimens indicated that the tumor was GA-FG (chief cell predominant type, GA-FG-CCP).

**Interventions:**

Endoscopic submucosal dissection (ESD) was performed. The histopathological examination of the ESD specimen revealed gastric hyperplasia of the fundic gland type around the adenocarcinoma cells.

**Outcomes:**

The surgical outcomes were good. The EGD showed a scar with no recurrence, and no symptoms were observed 1 year postoperatively during the follow-up.

**Conclusion:**

We present a rare case of a depressed lesion with a pathogenic expression suggesting gastric hyperplasia of the fundic gland type around the adenocarcinoma cells. Considering the origin of oxyntic mucosa, we consider that it may develop into GA-FG. To understand this issue better, similar cases should be monitored in the future.

## 1 Introduction

Gastric adenocarcinoma of the fundic gland type (GA-FG) is a novel entity, first proposed in 2010 by Ueyama.^[1]^ While before it, Tsukamoto^[2]^ defined a case as gastric adenocarcinoma with chief cell differentiation in 2007. Distinct from traditional intestinal and diffuse gastric carcinomas,^[3]^ GA-FG is a well-differentiated neoplasm with unclear etiopathogenesis and has a good prognosis. In previous reports, GA-FGs were found to be small tumors <1 cm in diameter, developing in the upper and middle third of the stomach.^[1,4–7]^ Several researchers have classified them into 3 types: elevated, flat, and depressed.^[1,5]^ Recently, we encountered a very rare case of a depressed lesion of GA-FG with gastric hyperplasia of the fundic gland type around the adenocarcinoma cells. Herein, we report this case, along with a brief review of the literature.

## 2 Case report

**Figure 1:**
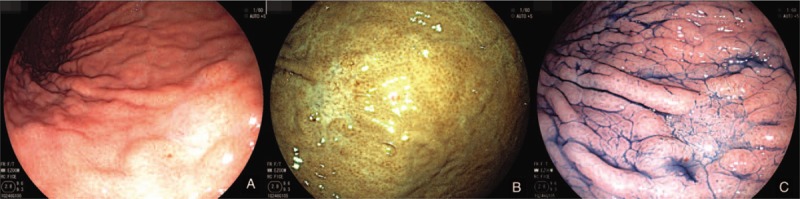
Endoscopic views of a lesion in the greater curvature. A and B: White-light and FICE (Fuji Intelligent Chromo Endoscopy) imaging show a whitish, depressed lesion in the middle third of the stomach. C: Indigo carmine spraying reveals the border of the lesion to be clear.

**Figure 2:**
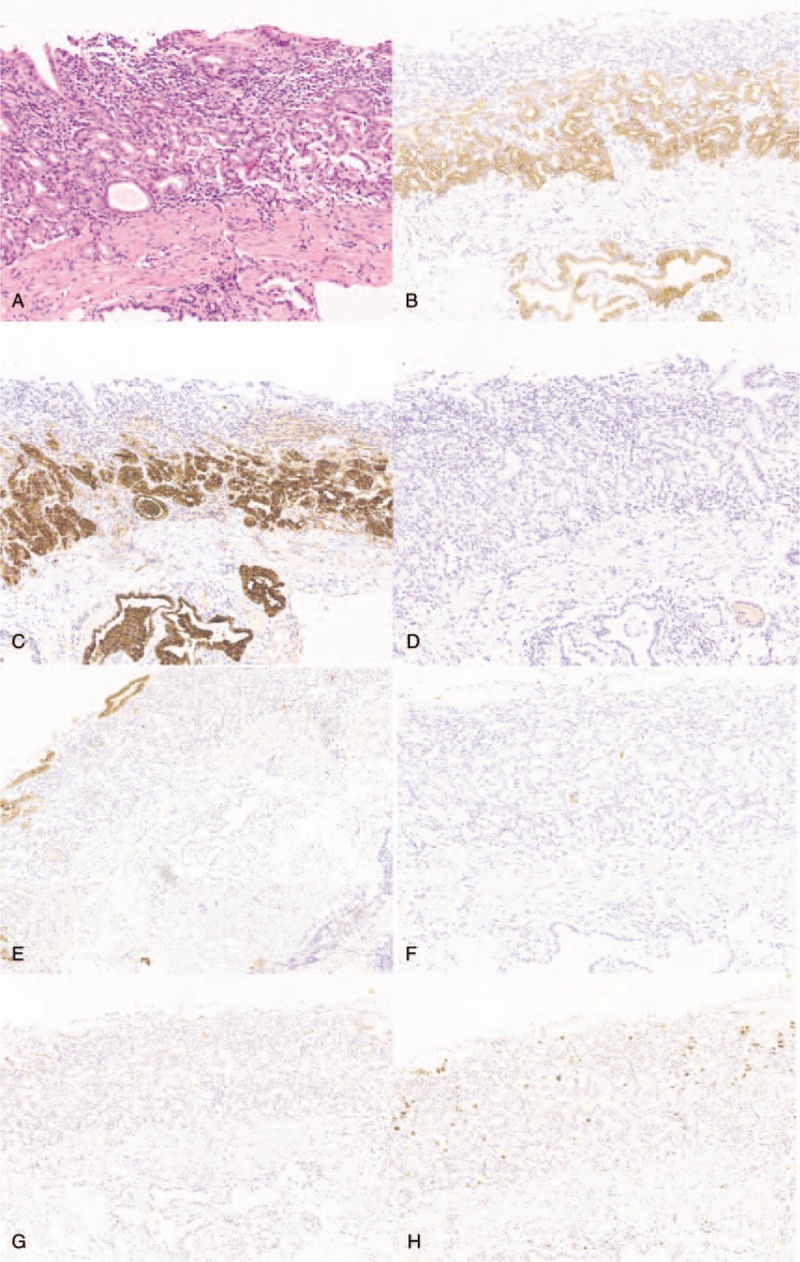
Histopathological and immunohistochemical findings of the first biopsy specimen. Tumor cells are diffusely positive for MUC6 (B) and pepsinogen I (C) and negative for H^+^/K^+^-ATPase (D), MUC5AC (E), and MUC2 (F). There is low expression of p53 protein (G) and low labeling index Ki-67 (H).

A 60-year-old man was referred to our hospital for abdominal distension. Endoscopy showed a whitish, depressed lesion measuring 1.2 cm in the greater curvature of the middle third of the stomach (Fig. 1). Histological examination of the biopsy specimens obtained from the lesion showed a well-differentiated tubular adenocarcinoma that mimicked the fundic glands and was positive for *Helicobacter pylori*. Some portion of the tumor surface was covered by non-atypical foveolar epithelium. Immunohistochemical examination revealed that the neoplastic glands were positive for mucin 6 (MUC6) and pepsinogen I, were negative for H^+^/K^+^-ATPase, MUC2, and MUC5AC, and had a low expression of p53 protein and low labeling index Ki-67 (<5%) (Fig. 2). These findings are characteristic of chief cell predominant type of GA-FG (GA-FG-CCP).

**Figure 3:**
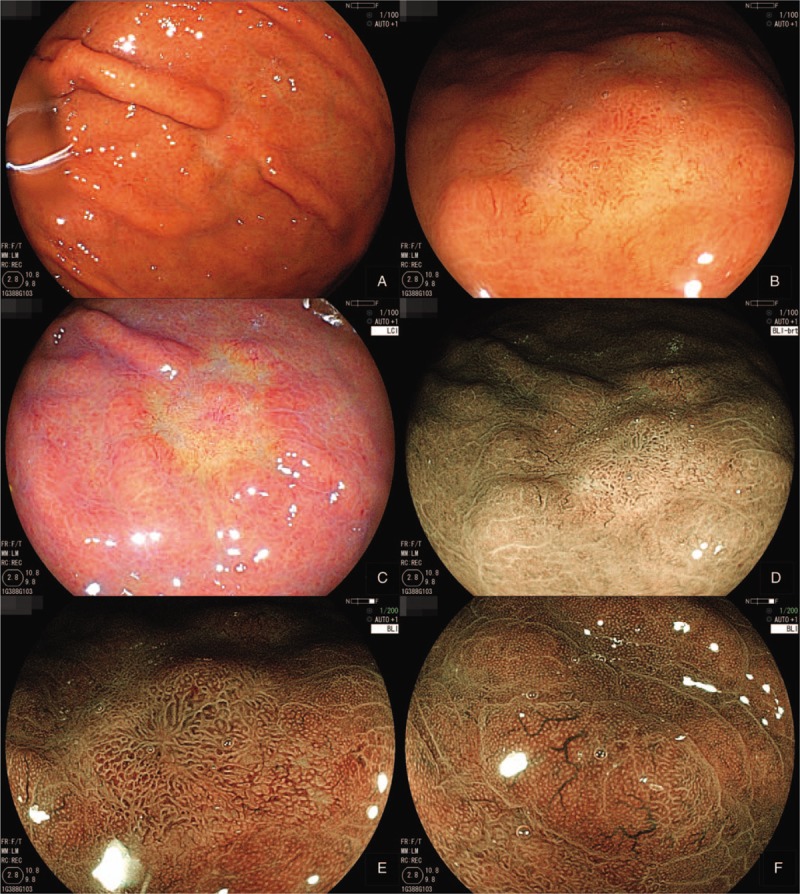
Magnifying endoscopy findings using LCI and BLI systems. A–D: A relatively regular micro-surface pattern is seen. E and F: BLI imaging shows vascular growth with dilated calibers and branching architecture, covered by a non-neoplastic, mildly enlarged foveolar epithelium.

Subsequently, magnification endoscopy using linked color imaging (LCI) and blue laser imaging (BLI) systems showed a relatively regular micro-surface pattern, plus vascular growth showing dilated calibers and branching architecture. These were covered with a non-neoplastic, mildly enlarged foveolar epithelium as seen after biopsy (Fig. 3). Endoscopic ultrasonography (EUS) findings revealed a hypoechoic mass (5 mm × 2 mm) located in the first layer and slightly compressing the second layer. As the histopathology of a forceps biopsy specimen revealed GA-FGs, the endoscopic submucosal dissection (ESD) was performed.

**Figure 4:**
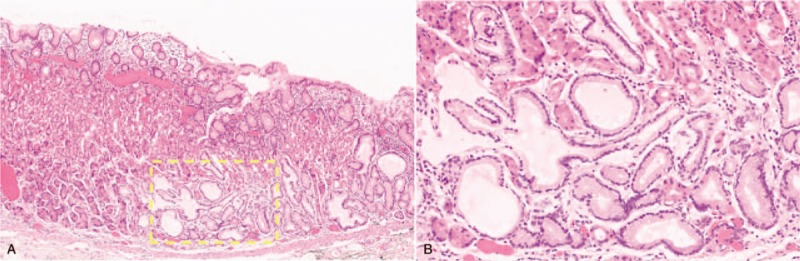
Hematoxylin and eosin staining shows that tumor cells are mainly localized in the mucosal layer.

**Figure 5:**
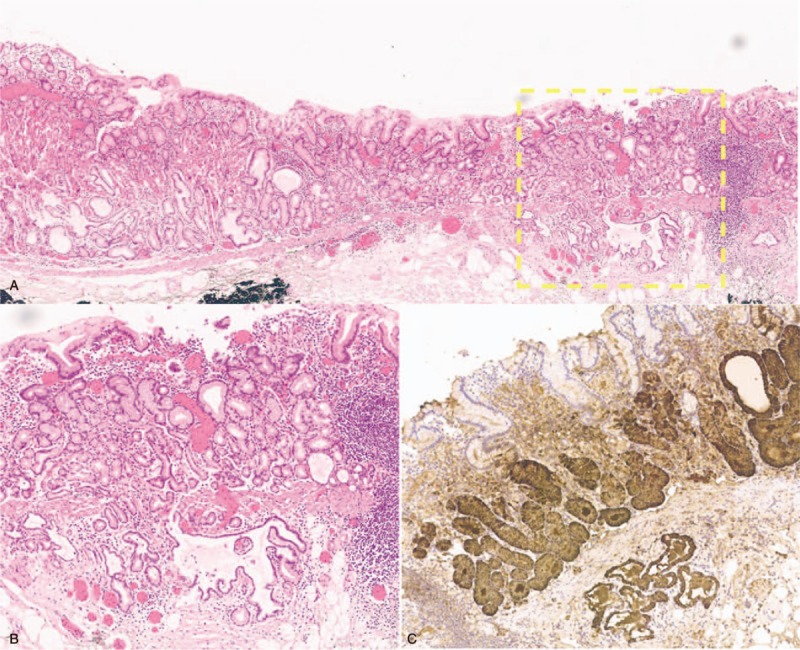
The tumor shows submucosal invasion, and the depth of invasion is 300 μm.

**Figure 6:**
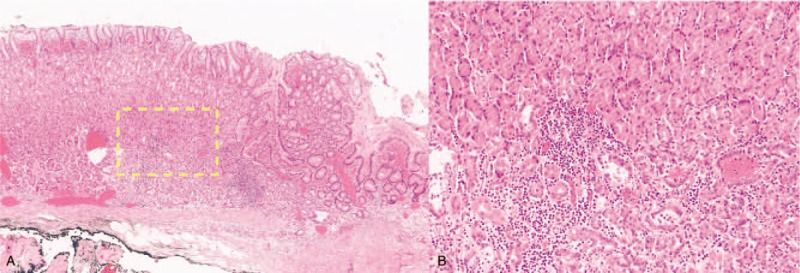
Hyperplasia-like segmental thickening glands are noted around the tumor cells.

**Figure 7:**
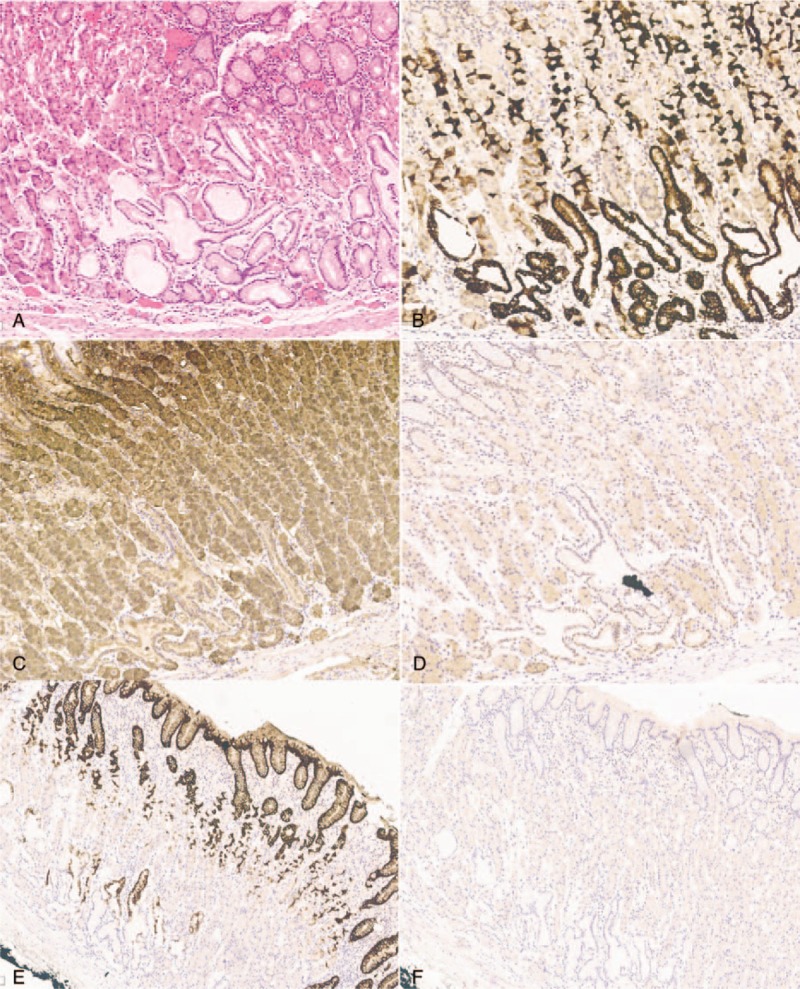
Histopathological and immunohistochemical findings of the endoscopic submucosal dissection specimen (A is HE). Tumor cells are diffusely positive for MUC6 (B) and pepsinogen I (C), and negative for H^+^/K^+^-ATPase (D), MUC5AC (E), and MUC2 (F).

Based on the ESD specimen, the tumor cells were localized in the deep mucosal layer, with invasion into the submucosal layer. Most of the tumor surface was covered with non-atypical foveolar epithelium (Fig. 4). The depth of submucosal invasion was 300 μm (Fig. 5). Hyperplasia-like segmental thickening glands were located around the tumor cells (Fig. 6). The adjacent oxyntic mucosa was normal without any intestinal metaplasia or atrophy. These findings were confirmed with the immunohistochemical examination of the first biopsy specimen. The neoplastic glands were diffusely and strongly reactive for MUC6 and pepsinogen I, respectively, and non-reactive for MUC2 and MUC5AC. No overexpression of the proliferation marker, Ki-67, and p53 protein was observed (Fig. 7). These results confirmed the diagnosis of GA-FG-CCP. At the same time, we tested the level of serum gastrin, and it was normal. The patient was followed up for 18 months. The esophagogastroduodenoscopy (EGD) showed a scar and no symptoms were observed postoperatively.

## 3 Discussion

GA-FGs are characterized as neoplastic lesions arising directly from gastric mucosa without intervening intestinal metaplasia. Most reports of GA-FG cases have been from Japan and Korea, with only a few reports from Western countries.^[4]^ It is often seen as covered by normal-colored or faded-whitish mucosa with vasodilatation or branched vessels on the tumor surface.^[1,4–7,11]^ Histologically, it can be divided into 3 subcategories: CCP type (approximately 99% of reported cases), parietal cell predominant type, and mixed phenotype.^[2,4,7,8]^ Findings in our case were typical, with the tumor observed in the middle third of the stomach, having a faded-whitish appearance, and with dilated vessels with branching architecture on the surface of the lesion. The tumor could be diagnosed as any of the following possibilities: undifferentiated type of gastric carcinoma, gastric lymphoma, focal atrophy, or GA-FG. The first 3 diseases can be distinguished easily based on the histological appearance.

GA-FGs arise from the lamina propria and spread laterally towards both the epithelium and submucosa. In previous reports, 63 (57%) of 111 reported cases of GA-FG were shown to have submucosal invasion,^[4]^ while 7 (6%) reported lymphovascular invasion.^[9–12]^ In some rare examples, Ueo et al,^[13]^ and Okumura et al,^[14]^ both reported a case of advanced-stage GA-FG with unusual clinicopathological features. But majority of studies^[8,10]^ have reported barely detectable endoscopic changes. Of the cases with follow-up data, Sato et al,^[8]^ observed no morphologic changes in the patient's tumor over a 12-year period. With that being said, the current evidence suggests that GA-FGs might be slow-growing tumors. As a result, most of GA-FGs have been treated by ESD or endoscopic mucosal resection,^[1,5–8,10,11]^ the other undergoing surgical resection,^[13,14]^ as conventional gastric cancers. The tumor in our case showed a submucosal invasion with a depth of 300 μm, and the Ki-67 index was low (<5%), consistent with the present data. And in our case, after a period of 18 months, the EGD results were normal, except for the presence of a scar. As yet, no specific treatment strategy has been established. Further detailed investigations are needed to determine whether GA-FGs exhibit high-grade malignancy.

In our case, according to the histopathological and immunohistochemical findings, we found gastric hyperplasia of the fundic glands positive for MUC6 and pepsinogen I, around the neoplastic cells. Chief cell- and parietal cell-hyperplasia may be found in association with fundic gland polyps and gastric hyperplasia of the fundic gland type,^[15–17]^ which are well-known changes known to occur when proton pump inhibitors (PPIs) are administered. Considering the fact that our patient had never been administered PPIs and that the level of serum gastrin was normal, the carcinogenesis remains undefined. However, these findings may explain the macroscopic depressed appearance. We speculate that the hyperplasia glands may develop into GA-FG. We should find more similar cases and observe the carcinogenesis of this special type of gastric carcinoma.

*H pylori* infection plays a major role in conventional adenocarcinoma.^[18]^ In previous reports, *H pylori* data were available in only 43 (39%) cases, and 17 (40%) were positive for infection.^[3]^ Our patient's endoscopy demonstrated an *H pylori* infection but no surrounding atrophy. This finding is consistent with that of most cases reported until now.^[1,4,5]^

In conclusion, we present a rare case of a depressed lesion with a pathogenic expression suggesting gastric hyperplasia of the fundic gland type around the adenocarcinoma cells. The patient needs to be closely followed up. Similar cases should be monitored in the future, to help clarify the pathogenesis of GA-FGs.

## Author contributions

**Endoscopist:** Xuesong Yang, Xiangchun Lin.

**Pathologist:** Yubo Ren.

**Patient management:** Lei Zhang, Peng Bai, Ranran Zeng

**Projection:** Li Wang

**Investigation:** Xin Li, Yan Wang, Yiming Ding

**Writing – original draft:** Xue Fan.

**Writing – review and editing:** Xue Fan, Xiangchun Lin.
